# Factors Associated With Continuum of Acute to Postacute Care in Stroke

**DOI:** 10.1155/srat/7409250

**Published:** 2026-07-24

**Authors:** Charmi Kanani, Julia Mitchell, Amit Kumar, Amol M. Karmarkar

**Affiliations:** ^1^ Department of Physical Medicine and Rehabilitation, and Center for Rehabilitation Science and Engineering, School of Medicine, Virginia Commonwealth University, Richmond, Virginia, USA, vcu.edu; ^2^ Sheltering Arms Institute, Richmond, Virginia, USA; ^3^ School of Medicine, Virginia Commonwealth University, Richmond, Virginia, USA, vcu.edu; ^4^ Department of Physical Therapy, University of Utah, Salt Lake City, Utah, USA, utah.edu

**Keywords:** continuum of care, postacute care, stroke, variation

## Abstract

**Background:**

Acute to postacute care continuum for individuals with stroke significantly influences functional recovery, quality of life, and healthcare utilization. Prior studies have focused largely on older adults with stroke, limiting generalizability for the younger population.

**Objective:**

This study is aimed at identifying patient‐ and hospital‐level factors associated with postacute discharge destination following acute stroke hospitalization.

**Design:**

This study is a retrospective cohort study.

**Setting:**

This study was set in acute care hospitals in Virginia (2017–2021).

**Participants:**

Participants are adults ≥ 18 years hospitalized with a primary diagnosis of ischemic or hemorrhagic stroke identified in the Virginia All‐Payer Claims Database.

**Interventions:**

There were no interventions applicable.

**Main Outcomes:**

Postacute care discharge destination was categorized as home, home health, skilled nursing facility (SNF), inpatient rehabilitation facility (IRF), or other. Patient‐level variables included age, sex, race, insurance, comorbidity burden, stroke type, length of stay, and hospital‐acquired complications. Facility‐level variables included hospital size, ownership, stroke center status, and county‐level clinical care rankings. Multivariable logistic regression models estimated associations between patient and hospital factors and postacute care discharge destination.

**Results:**

Older age, higher comorbidity burden, presence of hospital‐acquired complications, and longer length of stay were associated with increased likelihood of discharge to post‐acute care, particularly IRF and SNF. Male patients were less likely than females to be discharged to home health (OR = 0.90, 95*%*CI = 0.85–0.95) or to SNF (OR = 0.91, 95*%*CI = 0.87–0.96). Black patients had higher odds of discharge to home health (OR = 1.13, 95*%*CI = 1.05–1.22), IRF (OR = 1.10, 95*%*CI = 1.02–1.19), and SNF (OR = 1.13, 95*%*CI = 1.05–1.21) compared with White patients. Medicaid beneficiaries had higher odds of discharge to post‐acute care settings compared with commercially insured patients, including home health (OR = 1.34, 95*%*CI = 1.17–1.54), IRF (OR = 1.20, 95*%*CI = 1.05–1.38), and SNF (OR = 1.17, 95*%*CI = 1.02–1.34).

**Conclusions:**

Postacute care after stroke is associated with both clinical complexity and sociodemographic factors. These findings highlight potential differences and variations in postacute care and support the need for standardized discharge processes that can integrate medical and social determinants of health to optimize long‐term recovery.

## 1. Introduction

Stroke remains one of the leading causes of death and long‐term disability in the United States [1]. In the “Stroke Belt” states, defined as spanning across the southeastern United States, including Virginia, the burden of cerebrovascular disease is particularly pronounced, with stroke mortality rates estimated to be 20%–30% higher than the national average [2]. Contributing factors include higher rates of hypertension, diabetes, obesity, and tobacco use, as well as socioeconomic and healthcare access disparities that disproportionately affect medically underserved rural communities [2]. Many stroke survivors experience lasting and residual deficits in motor function, cognition, communication, and activities of daily living, which often necessitate postacute rehabilitative services to optimize recovery and functional independence [3–5]. Given these ongoing needs, a continuum of care frequently involves a transition from acute hospitalization to postacute care settings such as inpatient rehabilitation facilities (IRFs), skilled nursing facilities (SNFs), home health agencies (HHAs), or long‐term acute care hospitals (LTCHs) [6].

The clinical decision‐making process regarding postacute care discharge destination after stroke hospitalization play a critical role in determining patient recovery trajectories [7]. Placement in postacute care settings like IRFs offers patients structured, multidisciplinary, intense rehabilitation services, caregiver education, and discharge planning support not typically available when patients are directly discharged home [8]. However, the availability and geographic distribution of IRFs is limited, and access to specialized rehabilitation services remains a substantial barrier for patients living in rural communities, where transportation challenges, workforce shortages, and longer travel distances may delay or restrict access to postacute stroke care [9]. According to a 2025 Medicare Payment Advisory Commission (MedPAC) report, there are only 1206 IRFs across the United States with only 155 in a rural regions [10]. These disparities in rehabilitation infrastructure may contribute to geographic variation in discharge patterns and downstream stroke recovery outcomes. Numerous studies have demonstrated that stroke survivors who receive inpatient rehabilitation services experience better functional outcomes and lower long‐term disability compared to those discharged home with minimal services [11–13]. Additionally, evidence comparing IRF and SNF care shows that patients treated in IRFs tend to have greater functional improvement, higher rates of return to the community, and lower mortality and readmission rates, even after adjusting for baseline patient complexity [14]. Despite this, a large proportion of stroke patients in the United States are discharged home directly from acute care, even when their clinical status may warrant more intensive rehabilitation [15, 16].

This lack of alignment between patient needs and postacute care has contributed to wide variation in postacute care utilization, costs, and health outcomes [9]. Recent trends highlight growing concerns about disparities in access to postacute rehabilitation services [17]. These shifts have raised concerns about both the efficiency of care transitions, with significant demographic and geographic disparities observed in who receives postacute rehabilitation and in which setting [9]. Emerging evidence suggests that discharge decisions following stroke are influenced by more than just clinical severity. Although stroke severity and functional status are key determinants of postacute care placement, sociodemographic factors, particularly age, also play a critical role [17, 18]. Although stroke incidence rises with age, stroke incidence in young adults has been steadily increasing since the 1980s [19]. Younger stroke survivors may face distinct rehabilitation needs due to higher baseline functional status, greater potential for recovery, and unique social responsibilities, which can influence the intensity, type, and duration of postacute rehabilitation required [20]. Other factors such as race/ethnicity, gender, insurance type, and social support play a significant role in determining access to postacute services [21, 22]. Additionally, nonclinical factors, including hospital‐level characteristics such as size, teaching status, and geographic location, may further influence discharge patterns, though research on these factors remains limited [14].

Discharge disposition following stroke hospitalization is an important determinant of rehabilitation intensity, continuity of care, and long‐term functional recovery. Despite documented geographic disparities in access to rehabilitation services, relatively little is known about how geographic influences discharge disposition following stroke hospitalization, particularly across diverse payer populations. Existing studies have relied heavily on Medicare cohorts and often lack sufficient representation of younger adults or commercially insured patients. The All‐Payer Claims Database (APCD) offers a unique opportunity to examine postacute discharge patterns across a wider and more diverse patient population, encompassing multiple payer types and care settings. Therefore, the primary objective of this study was to identify sociodemographic, clinical, and hospital‐level factors associated with postacute discharge destination following acute hospitalization for stroke, using comprehensive APCD data for Virginia.

## 2. Methods

### 2.1. Ethics and Resource Sharing Statement

The study was approved by the local institutional review board with a waiver of informed consent due to the use of secondary deidentified data. We also established a Data Use Agreement (DUA) to use the APCD. We followed the Strengthening the Reporting of Observational Studies in Epidemiology (STROBE) reporting guideline [23].

### 2.2. Study Design and Data Source

**Figure 1:**
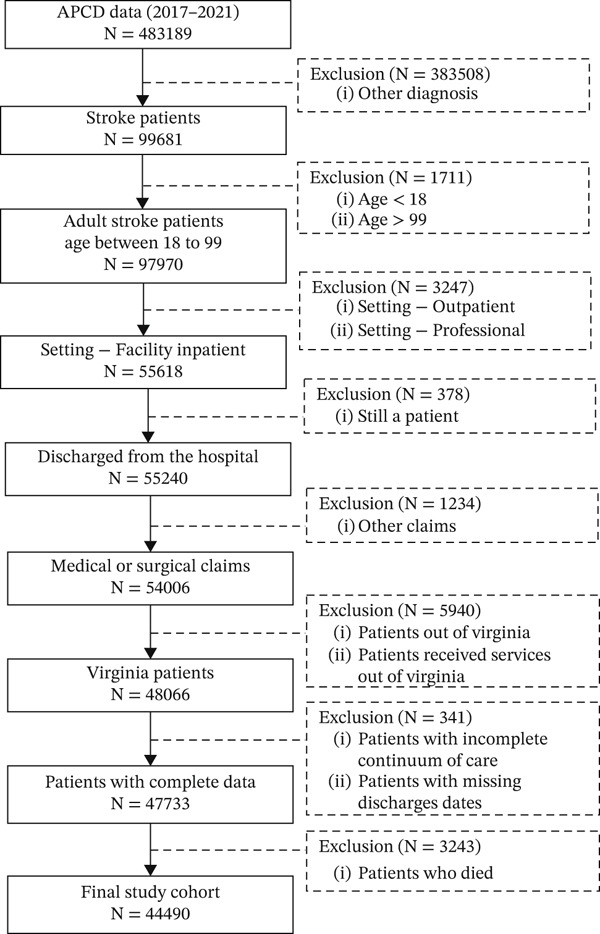
Cohort selection and derivation flowchart.

We conducted a retrospective cohort study with secondary analysis of data from the Virginia APCD between 2017 and 2021. Adults over age 18 with a primary diagnosis of stroke were identified using the International Classification of Diseases, Tenth Revision, Clinical Modification (ICD‐10 CM) Codes I60.x, I61.x, I62.x, and I63.x [1]. The study was restricted to index acute care hospitalizations in Virginia, defined as encounters listed as “Facility inpatient.” Only medical and surgical inpatient claims were included, and patients with incomplete care continuum trajectories, defined as those lacking discharge disposition information, missing postacute care claims within 30 days of hospital discharge, or with inconsistent admission/discharge dates, were excluded (Figure 1).

### 2.3. Data Cohort

**Figure 2:**
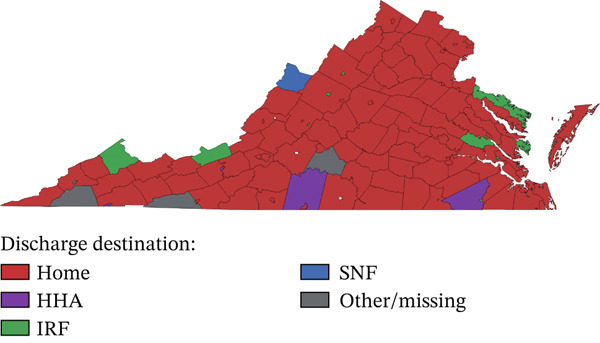
Most frequent postacute discharge destination. Abbreviations: HHA, home health agency; IRF, inpatient rehabilitation facility; SNF, skilled nursing facility.

**Figure 3:**
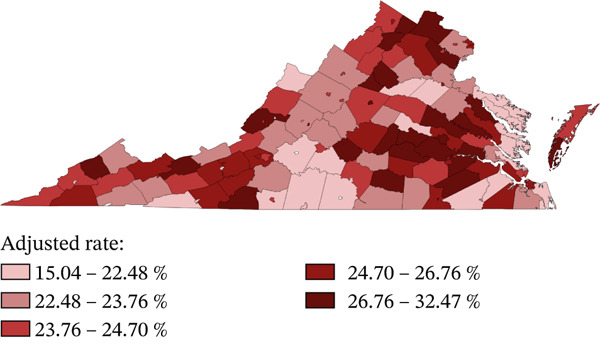
No postacute care distribution.

To ensure data quality, duplicate records were removed both at the row level and using encounter‐level identifiers, including admission and discharge dates, discharge status, and provider names. When discharge dates were missing, the subsequent admission date was substituted, and records marked as “still a patient” were excluded. For patients admitted in 2021 but discharged in 2022, the discharge year determined the study cohort, and such cases were attributed to 2021. The dataset was further restricted to Virginia residents treated by in‐state providers, with episodes starting with admission to nonacute facilities such as IRF, SNF, LTCH were excluded. Patient county of residence was determined using available county fields; if missing, the county was derived from the attributed primary care provider (PCP), and when the PCP was located out of state, the provider′s service location was used instead. Rurality was classified using Rural‐Urban Commuting Area (RUCA) codes, dichotomized as urban (1–7) or rural (8–9) [24]. County‐level clinical care quartiles were assigned using the 2019 County Health Rankings for pre‐2020 cases and the 2021 files [25]. At the county level, we calculated the adjusted rates of patients discharged directly to home without receiving postacute care. Additionally, we determined the adjusted rates of various discharge destinations for each county and identified the most common postacute care destination, which we then visualized. Variables used for adjusted rates include age, sex, race, resident area, Elixhauser score, presence of hospital acquired condition (HAC), insurance type, county‐level clinical care rank, socioeconomic rank, health outcomes rank, uninsured adults, population, severe housing problems, and number of SNFs, IRFs, and HHAs in the county (Figures 2 and 3).

### 2.4. Outcome Measures and Covariates

The primary outcome was postacute care discharge disposition, categorized into six mutually exclusive groups: home, home health, SNF, inpatient rehabilitation, expired, and other/missing. Other covariates included age, sex, race (White, Black, and other/missing), resident area (urban and rural), Elixhauser score, whether the patient had a HAC, hospital length of stay (LOS), type of stroke (hemorrhagic or ischemic), and insurance type (commercial/other, Medicare, and Medicaid). Facility‐level variables were incorporated by creating a provider–CCN crosswalk to link APCD providers with hospital information database. These included hospital bed size (≤ 200, 201–400, 401–600, and > 600), ownership type (for‐profit vs. nonprofit), and county‐level clinical care rank. Additional facility characteristics included system membership (whether the hospital belonged to a multihospital health system) and Council of Teaching Hospitals (COTH) membership. Profit status, as referenced in the results, was operationalized using ownership variable. RUCA classifications were also applied to provider locations to account for geographic context. Stroke center certification status was determined by merging with EMNet data, which identifies facilities certified or verified by the Joint Commission, Det Norske Veritas, Healthcare Facilities Accreditation Program, or local government agencies [26].

### 2.5. Statistical Analysis

We examined associations between patient and hospital characteristics and discharge destination following acute stroke hospitalization using a multilevel multinomial regression model. Discharge destination was modeled as a categorical outcome with multiple levels, using “home” as the reference category. A single multinomial model was fit in which each discharge setting was compared directly to the reference category. The model accounted for patient‐ and hospital‐level covariates and a random intercept for hospital (CCN) to account for clustering of patients within hospitals. The generalized logit link function was used and odds ratios and 95% confidence intervals were reported for each discharge category relative to home.

County‐level analyses were conducted separately from the patient‐level multilevel models and were intended to describe geographic variation in discharge patterns. The primary outcome for these analyses was the percentage of patients discharged to each postacute care setting within a county, calculated by aggregating patient‐level data by county and discharge destination. We then fit linear regression models using these county‐level percentages as outcomes, with predictors including aggregated demographic and clinical characteristics as well as county‐level characteristics. Model‐based predicted values were used to generate choropleth maps illustrating adjusted geographic variation in discharge destinations (Figures 2 and 3).

### 2.6. Patient Clinical and Sociodemographic Characteristics

Finally, patient‐level comorbidities were assessed using the Elixhauser Comorbidity Index, a validated tool that captures 31 comorbidity categories from administrative claims data to predict hospital outcomes such as mortality and readmission [27]. Hospital‐acquired complications (HACs) were identified using ICD‐10 diagnostic codes following established definitions for inpatient safety events [28]. In addition to comorbidities and HACs, patient sociodemographic characteristics, including age, sex, race/ethnicity, and residential location (urban vs. rural), were captured from administrative records to examine their association with postacute care utilization.

## 3. Results

Among 44,490 adults hospitalized for stroke in Virginia from 2017 to 2021, 31.9% were discharged home, 19.2% to HHA, 19.4% to IRF, 21.4% to SNFs, and 8.2% to the other/missing category. Use of HHA was higher after 2020 (23.8% and 22.5% in 2020 and 2021, respectively), whereas SNF discharges declined after 2020 (15.9% and 17.5% in 2020 and 2021, respectively).

Age distributions varied by discharge setting: patients ≤ 65 years accounted for 35.6% of home discharges but only 11.4% of SNF discharges, whereas patients ≥ 75 years comprised 68.0% of SNF discharges. Females represented 52.8% of the cohort and were more likely to be discharged to SNF (59.3%) or HHA (58.1%). Most patients were White (58.8%), urban residents (94.0%), and had 1–3 Elixhauser comorbidities (46.2%). Hemorrhagic strokes accounted for 14.8% of admissions. Medicare was the predominant insurance type (78.8%), followed by Medicaid (9.9%) and Commercial/Other (11.3%). Mean hospital LOS ranged from 3.9 days for patients discharged home to 8.5 days for SNF discharges (Table 1).

**Table 1:** Descriptive factors associated with postacute care discharge destinations.

Variable	Overall	Home	HHA	IRF	SNF	Other/missing
**Patient-level**						
Number of subject	44490	14187	8521	8646	9502	3634
Study year, *n* (%)						
2017	8599 (19.3)	2742 (19.3)	1449 (17.0)	1574 (18.2)	2185 (23.0)	649 (17.9)
2018	8648 (19.4)	2757 (19.4)	1458 (17.1)	1622 (18.8)	2145 (22.6)	666 (18.3)
2019	9172 (20.6)	2940 (20.7)	1663 (19.5)	1802 (20.8)	1999 (21.0)	768 (21.1)
2020	8898 (20.0)	2770 (19.5)	2031 (23.8)	1816 (21.0)	1512 (15.9)	769 (21.2)
2021	9173 (20.6)	2978 (21.0)	1920 (22.5)	1832 (21.2)	1661 (17.5)	782 (21.5)
Age, *n* (%)						
≤ 65	10820 (24.3)	5045 (35.6)	1961 (23.0)	2122 (24.5)	1079 (11.4)	613 (16.9)
66–74	11690 (26.3)	4423 (31.2)	2114 (24.8)	2557 (29.6)	1966 (20.7)	630 (17.3)
75+	21980 (49.4)	4719 (33.3)	4446 (52.2)	3967 (45.9)	6457 (68.0)	2391 (65.8)
Sex, *n* (%)						
Male	21001 (47.2)	7727 (54.5)	3571 (41.9)	4270 (49.4)	3871 (40.7)	1562 (43.0)
Female	23489 (52.8)	6460 (45.5)	4950 (58.1)	4376 (50.6)	5631 (59.3)	2072 (57.0)
Race, *n* (%)						
White	26152 (58.8)	7948 (56.0)	4774 (56.0)	5103 (59.0)	5962 (62.7)	2365 (65.1)
Black	8910 (20.0)	2254 (15.9)	1947 (22.8)	1962 (22.7)	2094 (22.0)	653 (18.0)
Others	9428 (21.2)	3985 (28.1)	1800 (21.1)	1581 (18.3)	1446 (15.2)	616 (17.0)
Resident area, *n* (%)						
Urban	41800 (94.0)	13365 (94.2)	8000 (93.9)	8134 (94.1)	8893 (93.6)	3408 (93.8)
Rural	2690 (6.0)	822 (5.8)	521 (6.1)	512 (5.9)	609 (6.4)	226 (6.2)
Elixhauser score, *n* (%)						
0	10094 (22.7)	2991 (21.1)	2198 (25.8)	2084 (24.1)	1836 (19.3)	985 (27.1)
1–3	20546 (46.2)	7688 (54.2)	3631 (42.6)	3664 (42.4)	3993 (42.0)	1570 (43.2)
3+	13850 (31.1)	3508 (24.7)	2692 (31.6)	2898 (33.5)	3673 (38.7)	1079 (29.7)
At least one HAC, *n* (%)						
Yes	612 (1.4)	73 (0.5)	98 (1.2)	119 (1.4)	233 (2.5)	89 (2.4)
No	43878 (98.6)	14114 (99.5)	8423 (98.9)	8527 (98.6)	9269 (97.5)	3545 (97.6)
LOS, mean (SD)	6.13 (10.3)	3.93 (8.13)	5.07 (5.44)	7.34 (8.57)	8.49 (10.9)	8.15 (20.9)
Type of stroke, *n* (%)						
Hemorrhagic	6585 (14.8)	1694 (11.9)	1041 (12.2)	1410 (16.3)	1463 (15.4)	977 (26.9)
Ischemic	37905 (85.2)	12493 (88.1)	7480 (87.8)	7236 (83.7)	8039 (84.6)	2657 (73.1)
Insurance type, *n* (%)						
Commercial/other	5047 (11.3)	2868 (20.2)	707 (8.3)	749 (8.7)	508 (5.3)	215 (5.9)
Medicaid	4396 (9.9)	1542 (10.9)	1015 (11.9)	949 (11.0)	563 (5.9)	327 (9.0)
Medicare	35047 (78.8)	9777 (68.9)	6799 (79.8)	6948 (80.4)	8431 (88.7)	3092 (85.1)

**Hospital-level**						
Profit status, *n* (%)						
Profit	6467 (14.5)	1951 (13.8)	1044 (12.3)	1675 (19.4)	1164 (12.3)	633 (17.4)
Nonprofit	38023 (85.5)	12236 (86.2)	7477 (87.7)	6971 (80.6)	8338 (87.8)	3001 (82.6)
Certified by the Joint Commission, Det Norske Veritas, Healthcare Facilities Accreditation Program, or local government, *n* (%)						
Yes	41135 (92.5)	13195 (93.0)	7905 (92.8)	8076 (93.4)	8652 (91.1)	3307 (91.0)
No	3355 (7.5)	992 (7.0)	616 (7.2)	570 (6.6)	850 (8.9)	327 (9.0)
Member of the COTH of the Association of American Medical Colleges, *n* (%)						
Yes	10190 (22.9)	3306 (23.3)	1725 (20.2)	2241 (25.9)	2043 (21.5)	875 (24.1)
No	34300 (77.1)	10881 (76.7)	6796 (79.8)	6405 (74.1)	7459 (78.5)	2759 (75.9)
System member, *n* (%)						
Yes	41354 (93.0)	13029 (91.8)	7951 (93.3)	8203 (94.9)	8743 (92.0)	3428 (94.3)
No	3136 (7.0)	1158 (8.2)	570 (6.7)	443 (5.1)	759 (8.0)	206 (5.7)
Number of beds, *n* (%)						
≤ 200	9105 (20.5)	3019 (21.3)	1953 (22.9)	1383 (16.0)	1999 (21.0)	751 (20.7)
201–400	15584 (35.0)	4748 (33.5)	3121 (36.6)	2808 (32.5)	3692 (38.9)	1215 (33.4)
401–600	6665 (15.0)	2202 (15.5)	1300 (15.3)	1435 (16.6)	1211 (12.7)	517 (14.2)
> 600	13136 (29.5)	4218 (29.7)	2147 (25.2)	3020 (34.9)	2600 (27.4)	1151 (31.7)
Clinical care quartile, *n* (%)						
1	17073 (38.4)	5729 (40.4)	3089 (36.3)	3497 (40.4)	3352 (35.3)	1406 (38.7)
2	19278 (43.3)	5963 (42.0)	3722 (43.7)	3886 (44.9)	4214 (44.3)	1493 (41.1)
3	6308 (14.2)	1963 (13.8)	1354 (15.9)	938 (10.8)	1497 (15.8)	556 (15.3)
4	1831 (4.1)	532 (3.8)	356 (4.2)	325 (3.8)	439 (4.6)	179 (4.9)

Abbreviations: COTH, Council of Teaching Hospitals; HAC, hospital acquired condition; HHA, home health agency; IRF, inpatient rehabilitation facility; LOS, length of stay; SNF, skilled nursing facility.

Compared with patients ≤ 65 years, those aged 66–74 had slightly increased odds of SNF discharge (OR = 1.13, 95*%*CI = 1.04–1.24), whereas patients ≥ 75 years had higher odds of discharge to HHA (OR = 1.25, 95*%*CI = 1.15–1.36), IRF (OR = 1.16, 95*%*CI = 1.07–1.26), SNF (OR = 1.52, 95*%*CI = 1.40–1.66), and combined other/missing discharge category (OR = 1.41, 95*%*CI = 1.26–1.58). Male patients were less likely than females to be discharged to HHA (OR = 0.90, 95*%*CI = 0.85–0.95) or SNF (OR = 0.91, 95*%*CI = 0.86–0.96). Compared with White patients, Black patients had higher odds of discharge to HHA (OR = 1.13, 95*%*CI = 1.05–1.22), IRF (OR = 1.10, 95*%*CI = 1.02–1.19), and SNF (OR = 1.12, 95*%*CI = 1.04–1.20).

Clinical factors were also associated with discharge destination. Patients with Elixhauser scores ≥ 3 had higher odds of SNF discharge (OR = 1.18, 95*%*CI = 1.08–1.30). Hemorrhagic stroke was not associated with discharge to HHA, IRF, or SNF. Longer hospital LOS was associated with higher odds of discharge to HHA (OR = 1.01, 95*%*CI = 1.00–1.01), IRF (OR = 1.02, 95*%*CI = 1.02–1.03), SNF (OR = 1.03, 95*%*CI = 1.02–1.03), and combined other/missing discharge category (OR = 1.02, 95*%*CI = 1.02–1.03) (Table 2).

**Table 2:** Adjusted analysis of postacute care discharge destinations.

Variable	HHA	IRF	SNF	Other/missing
Number of patients	8521	8646	9502	3634
**Patient-level**				
Study year				
2017	0.91 (0.83–1.01)	0.97 (0.88–1.07)	1.05 (0.95–1.16)	0.98 (0.85–1.12)
2018	0.92 (0.83–1.01)	0.99 (0.89–1.09)	1.05 (0.95–1.15)	0.98 (0.86–1.12)
2019	0.93 (0.84–1.02)	0.99 (0.90–1.09)	1.01 (0.92–1.11)	1.00 (0.88–1.14)
2020	1.00	1.00	1.00	1.00
2021	0.96 (0.88–1.04)	0.98 (0.90–1.07)	1.01 (0.93–1.10)	0.98 (0.87–1.10)
Age				
≤ 65	1.00	1.00	1.00	1.00
66–74	1.05 (0.96–1.15)	1.06 (0.97–1.16)	**1.13 (1.04–1.24)**	1.02 (0.90–1.15)
75+	**1.25 (1.15–1.36)**	**1.16 (1.07–1.26)**	**1.52 (1.40–1.66)**	**1.41 (1.26–1.58)**
Sex				
Male	**0.90 (0.85–0.95)**	0.97 (0.92–1.02)	**0.91 (0.86–0.96)**	**0.92 (0.86–0.99)**
Female	1.00	1.00	1.00	1.00
Race				
White	1.00	1.00	1.00	1.00
Black	**1.13 (1.05–1.22)**	**1.10 (1.02–1.19)**	**1.12 (1.04–1.20)**	1.04 (0.94–1.15)
Others	**1.10 (1.01–1.21)**	1.00 (0.91–1.09)	1.01 (0.93–1.11)	1.01 (0.90–1.14)
Resident area				
Urban	0.99 (0.88–1.12)	0.99 (0.88–1.12)	1.00 (0.88–1.12)	1.00 (0.85–1.17)
Rural	1.00	1.00	1.00	1.00
Elixhauser score				
0	1.00	1.00	1.00	1.00
1–3	0.96 (0.88–1.06)	0.96 (0.87–1.05)	1.03 (0.94–1.13)	0.94 (0.83–1.06)
3+	1.06 (0.97–1.17)	1.08 (0.98–1.19)	**1.18 (1.08–1.30)**	1.01 (0.89–1.16)
At least one HAC				
Yes	1.10 (0.86–1.41)	1.11 (0.87–1.41)	**1.31 (1.05–1.64)**	1.32 (0.98–1.78)
No	1.00	1.00	1.00	1.00
LOS	**1.01 (1.00–1.01)**	**1.02 (1.02–1.03)**	**1.03 (1.02–1.03)**	**1.02 (1.02–1.03)**
Type of stroke				
Hemorrhagic	1.02 (0.93–1.10)	1.03 (0.95–1.11)	1.05 (0.97–1.13)	**1.32 (1.20–1.47)**
Ischemic	1.00	1.00	1.00	1.00
Insurance type				
Commercial/other	1.00	1.00	1.00	1.00
Medicaid	**1.32 (1.16–1.51)**	**1.19 (1.04–1.37)**	1.14 (0.99–1.30)	**1.25 (1.04–1.51)**
Medicare	**1.28 (1.13–1.44)**	**1.22 (1.08–1.37)**	**1.24 (1.10–1.39)**	**1.27 (1.07–1.49)**

**Hospital-level**				
Profit status				
Profit	1.00	1.00	1.00	1.00
Nonprofit	1.04 (0.89–1.21)	0.88 (0.71–1.10)	1.03 (0.85–1.26)	0.93 (0.77–1.13)
Certified by the Joint Commission, Det Norske Veritas, Healthcare Facilities Accreditation Program, or local government				
Yes	0.99 (0.85–1.17)	1.01 (0.80–1.26)	0.89 (0.72–1.09)	0.94 (0.77–1.14)
No	1.00	1.00	1.00	1.00
Member of the COTH of the Association of American Medical Colleges				
Yes	1.00 (0.77–1.29)	1.01 (0.67–1.52)	1.00 (0.69–1.46)	0.97 (0.73–1.28)
No	1.00	1.00	1.00	1.00
System member				
Yes	1.06 (0.84–1.35)	1.14 (0.78–1.66)	1.00 (0.72–1.39)	1.08 (0.81–1.43)
No	1.00	1.00	1.00	1.00
Number of beds				
≤ 200	1.00	1.00	1.00	1.00
201–400	1.00 (0.87–1.15)	1.09 (0.87–1.35)	1.05 (0.86–1.28)	1.02 (0.86–1.22)
401–600	0.96 (0.80–1.15)	1.04 (0.79–1.38)	0.93 (0.72–1.19)	0.94 (0.76–1.16)
> 600	0.93 (0.72–1.22)	1.08 (0.71–1.64)	0.98 (0.67–1.42)	1.01 (0.75–1.36)
Clinical care quartile				
1	1.00	1.00	1.00	1.00
2	1.04 (0.91–1.19)	0.98 (0.80–1.20)	1.07 (0.89–1.28)	1.00 (0.86–1.16)
3	1.03 (0.89–1.20)	0.93 (0.75–1.16)	1.07 (0.88–1.30)	1.05 (0.88–1.26)
4	1.02 (0.83–1.27)	0.99 (0.74–1.33)	1.09 (0.83–1.42)	1.11 (0.86–1.43)

*Note:* Significant ORs are bolded.

Abbreviations: COTH, Council of Teaching Hospitals; HAC, hospital acquired condition; HHA, home health agency; IRF, inpatient rehabilitation facility; LOS, length of stay; SNF, skilled nursing facility.

Insurance type was significantly associated with postacute care use. Compared with commercially insured patients, those with Medicaid had higher odds of discharge to HHA (OR = 1.32, 95*%*CI = 1.16–1.51), IRF (OR = 1.19, 95*%*CI = 1.04–1.37), and combined other/missing discharge category (OR = 1.25, 95*%*CI = 1.04–1.51). Medicare beneficiaries had increased odds of discharge across all postacute care settings. Rural residence was not independently associated with discharge destination in adjusted analyses; however, only 6% of the cohort was classified as rural. Similarly, most hospital‐level characteristics, including hospital size, stroke certification status, and system membership, were not significantly associated with discharge disposition. Figures 2 and 3 demonstrated notable county‐level variation in post‐acute care utilization patterns across Virginia despite the absence of statistically significant associations between rural residence and discharge destination in adjusted analyses.

## 4. Discussion

We found that multiple patient‐level factors were associated with postacute discharge destination following stroke hospitalization in Virginia. Age, sex, race, comorbidity burden, stroke type, insurance status, and hospital characteristics all contributed to variation in postacute care utilization. Our analysis using the APCD from Virginia uniquely extends prior work by incorporating a broader payer mix beyond Medicare, allowing for a more representative picture of discharge patterns across a diverse population. Although older adults were more frequently discharged to institutional postacute care settings, stroke incidence among adults younger than 65 has been increasing [19]. Younger stroke survivors often have higher baseline functional status, greater potential for recovery, and unique social responsibilities, including employment and caregiving [20]. If these patients do not receive intensive postacute care, they are at risk for long‐term residual deficits in mobility, cognition, and mental health problems, which can significantly affect independence, quality of life, and long‐term socioeconomic outcomes [29]. Incorporating younger adults in our analysis highlights potential gaps in postacute care utilization and underscores the importance of ensuring that rehabilitation services meet the needs of this growing population.

Our analysis revealed that older patients were more likely to be discharged to institutional postacute care settings such as SNFs (patients ≥ 75 years had 52% higher odds) or IRFs (16% higher odds). This aligns with existing literature indicating that older adults often require more extended postacute care due to increased medical complexity and functional decline [30]. Similarly, our data showed that longer hospital stays were associated with a higher likelihood of discharge to postacute care settings, including home health, IRF, and SNF. Extended hospital stays often reflect more complicated hospital courses or slower recovery trajectories, necessitating continued care in postacute settings [7, 14]. Patients with greater comorbidity burdens (Elixhauser ≥ 3: 18% higher odds for SNF) and those experiencing HACs (31% higher odds for SNF) also showed similar patterns, reinforcing the central role of medical complexity in determining the need for ongoing skilled care. Comorbidities significantly impact postacute outcomes, with patients having multiple comorbidities more likely to experience complications and require extended rehabilitation [31].

We identified sociodemographic differences in discharge disposition. Male patients were less likely than females to be discharged to home health (10% less likely) or SNF (9% less likely), consistent with prior research suggesting that women are more likely to receive postacute services, potentially due to differences in social support networks, caregiver availability, or healthcare‐seeking behaviors [32]. Black patients were more likely than White patients to be discharged to home health (13% more likely), IRF (10% more likely), and SNF (12% more likely), and patients categorized as “other” race were more likely to be discharged to home health (10% more likely) (Table 2). This contrasts with earlier studies reporting underutilization of postacute care among racial and ethnic minorities [33]. The divergence may reflect the broader patient population captured in the APCD, regional variation in service delivery, or differences in insurance coverage that affect access to postacute care. Overall, these findings indicate that both sex and race are important factors influencing postacute care utilization, shaped by a combination of social, geographic, and structural factors.

Insurance coverage type significantly influences discharge destination to postacute care settings. Patients with Medicaid were more likely than those with commercial/other insurance to be discharged to home health (32% more likely), IRF (19% more likely), and other discharge destinations (25% more likely). Medicare beneficiaries similarly had increased odds of discharge across all postacute care settings (Table 2). This finding contrasts with prior research reporting lower odds of postacute care among Medicaid patients [34, 35]. The divergence may reflect the broader APCD population, regional policy factors, or hospital‐level practices. These findings underscore the importance of payer type in shaping access to rehabilitation services and highlight the need for policy reforms that ensure equitable access across insurance groups.

Although adjusted patient‐level analyses did not demonstrate statistically significant associations between rural residence and discharge destination, descriptive county‐level analyses revealed notable geographic heterogeneity in postacute care utilization patterns across Virginia. Several factors may explain this apparent discrepancy. First, county‐level dichotomized RUCA classifications may not fully capture localized barriers to rehabilitation access, including transportation limitations, rehabilitation workforce shortages, facility distribution, and travel distance to specialized postacute care services. Second, descriptive geographic variation may reflect differences in regional healthcare infrastructure or referral patterns that were not fully accounted for in adjusted models. Finally, the relatively small proportion of patients classified as rural may have limited statistical power to detect meaningful geographic differences.

Discharge destination following acute stroke hospitalization has important clinical implications because postacute care settings differ substantially in rehabilitation intensity, care coordination, and available support services. The associations observed between medical complexity, insurance type, and discharge disposition may help clinicians identify patients likely to require higher levels of postacute support earlier during hospitalization. Additionally, the geographic variation observed in postacute care utilization patterns highlights the importance of healthcare infrastructure and access to rehabilitation services within stroke systems of care. Improved understanding of factors associated with discharge disposition may facilitate more individualized discharge planning and support efforts to optimize functional recovery following stroke.

Taken together, our findings suggest that discharge decisions after stroke hospitalization are linked to a complex interplay of clinical, demographic, and structural factors. As expected, older age, higher burden of comorbidities, and in‐hospital complications were associated with a greater likelihood of discharge to institutional postacute care settings such as IRF or SNF. However, the persistent influence of race and insurance status on discharge patterns may highlight potential inequities in how postacute services are allocated. For example, patients from racial or ethnic minority groups or those with Medicaid or no insurance may face barriers to accessing higher‐intensity rehabilitation, independent of their clinical needs. This raises concerns about the standardization of discharge practices and whether patients are consistently matched to the care setting that best supports their recovery. The observed variability in discharge destinations may contribute to disparities in functional outcomes and long‐term recovery, as access to timely and appropriate postacute care is a key determinant of rehabilitation success. These findings underscore the importance of identifying both patient‐level factors (e.g., clinical severity, social support, and insurance coverage) and hospital‐level factors (e.g., bed availability, institutional policies, and regional care networks) that shape discharge decisions. Ultimately, understanding these determinants is critical for developing policies and payment models that promote equitable access to adequate and appropriate post‐acute care, ensure care aligns with patient needs, and reduce disparities in stroke care, and optimize long‐term stroke recovery across diverse populations [9].

## 5. Limitations

This study has several limitations. First, APCD data lack key clinical variables such as the NIH Stroke Scale, functional assessments (e.g., modified Rankin Scale), and imaging findings, all of which are essential for understanding stroke severity and guiding discharge decisions. The absence of these measures limits our ability to adjust for clinical acuity. Second, misclassification of diagnoses, comorbidities, and complications is possible due to reliance on ICD‐10 coding, which may vary across providers and institutions. Claims data do not distinguish between preexisting conditions and complications developing during hospitalization unless explicitly coded, which may lead to underestimation or overestimation of certain clinical factors. Administrative claims data lack detailed measures of stroke severity and functional status, which are important determinants of discharge disposition. Third, we limited healthcare utilization only within Virginia. Also, patients receiving care outside state borders or those moving between states may have incomplete data, which affects generalizability. Additionally, patterns of postacute care availability, insurance coverage, and healthcare infrastructure in Virginia may differ from other regions, limiting external validity. Potential geographic misclassification may have occurred when county assignment relied on PCP attribution, and dichotomized county‐level RUCA classifications may not adequately capture geographic heterogeneity. Several limitations related to the operationalization of rurality should be considered when interpreting these findings. The dichotomized county‐level RUCA classification, although consistent with previously published approaches, may have reduced the ability to detect more nuanced geographic variation in postacute care utilization. County‐level rurality measures may also fail to capture important within‐county heterogeneity in healthcare access, rehabilitation infrastructure, transportation availability, and distance to specialized postacute care services. Additionally, only 6% of the cohort was classified as rural, which may have limited statistical power to detect meaningful geographic differences in discharge disposition. Fourth, important social determinants of health, including caregiver availability, socioeconomic status, education level, housing stability, transportation, and patient preferences, are not captured in APCD data and most likely influence the postacute care discharge planning process. Residual confounding from unmeasured social determinants of health is possible. These unmeasured factors may contribute to observed differences by race, insurance status, or geography. Fifth, facility‐level characteristics obtained from external datasets (e.g., EMNet) may contain reporting lags or inaccuracies, and not all facility attributes relevant to discharge processes, such as rehabilitation capacity, staffing ratios, or discharge planning resources, were available. Finally, because this is an observational study, associations cannot be interpreted as causal.

## 6. Conclusions and Implications

This study adds to the growing body of literature on stroke acute to postacute care continuum by demonstrating how sociodemographic and insurance‐related factors intersect with clinical characteristics to influence postacute care utilization. First, APCD data provide a valuable platform to evaluate stroke acute to postacute care transitions in populations not captured by Medicare‐only analyses. Second, the results underscore the need for standardized, equitable discharge assessment tools that integrate both clinical severity and social determinants of health. Third, the role of payer type highlights how insurance design and reimbursement structures may drive inequities in access to post‐acute care and rehabilitation. As payment models increasingly shift toward value‐based care, where reimbursement is tied to patient outcomes rather than volume of services, understanding these dynamics is critical [34, 35]. In a value‐based care framework, ensuring that patients are discharged to the most appropriate postacute setting is not only a matter of equity but also reflects care efficiencies that all the healthcare systems and providers strive for. Future research should incorporate measures of stroke severity and functional outcomes, evaluate patient and caregiver perspectives, and assess the impact of discharge destination on long‐term recovery, functional independence, and quality of life, thereby informing strategies that align clinical need with efficient, equitable resource allocation, as well as policies that ensure adequate and appropriate allocation of postacute care resources to optimize recovery for all stroke survivors. Future research should also incorporate more granular geographic measures, detailed stroke severity and functional status data, and multilevel analytic approaches to further clarify the complex factors influencing postacute discharge disposition following stroke hospitalization. Additional investigation into regional rehabilitation availability and barriers to postacute care access may also help inform strategies to optimize recovery and continuity of care for stroke survivors.

## Funding

This work was supported by the Commonwealth Neurotrauma Initiative (CNI): Transitions and Disparities in Care and Outcomes (TDCO) for Neurotrauma, A262‐90012, and the VCU C. Kenneth and Dianne Wright Center for Clinical and Translational Research, UM1TR004360.

## Conflicts of Interest

The authors declare no conflicts of interest.

## Data Availability

Due to Data Use Agreement restrictions, we cannot make the data available, however, it can be obtained from Virginia All‐Payer Claims Database (APCD). Virginia Health Information is available at: https://www.vhi.org/apcd.
